# Measuring training effectiveness of laboratory biosafety program offered at African Center for Integrated Laboratory Training in 22 President’s Emergency Plan for AIDS Relief supported countries (2008–2014)

**DOI:** 10.1186/s41182-023-00557-1

**Published:** 2023-11-21

**Authors:** Ritu Shrivastava, Thomas Stevens, Larry Westerman, David Bressler, Elsie van Schalkwyk, Cristina Bressler, Ken Ugwu, Christina Mwangi, Joel Peter Opio, Joseph Nkodyo, Jane W. Mwangi, Monte D. Martin, Shanna Nesby-O’Dell

**Affiliations:** 1 Centers for Disease Control and Prevention, Atlanta, USA; 2 Centers for Disease Control and Prevention, Lusaka, Zambia; 3 African Center for Integrated Laboratory Training, Johannesburg, South Africa; 4 Government of Canada, Ottawa, Canada; 5 Centers for Disease Control and Prevention, Kampala, Uganda; 6 Ministry of Health, Kampala, Uganda; 7 Centers for Disease Control and Prevention, Nairobi, Kenya

## Abstract

**Introduction:**

The African Center for Integrated Laboratory Training (ACILT) in Johannesburg, South Africa offered a laboratory biosafety program to improve laboratory biosafety practices in 22 President’s Emergency Plan for AIDS Relief (PEPFAR) supported countries. This manuscript evaluates the transference of newly gained knowledge and skills to the participants’ place of employment for HIV and TB diagnostic laboratory programs. It also serves as a follow-on to a previously published manuscript that measured training effectiveness for all courses offered at ACILT.

**Methods:**

ACILT offered 20 Laboratory Biosafety and Infrastructure courses (2008–2014), also referred as biosafety course/course comprising of 14 core laboratory safety elements to 402 participants from 22 countries. In 2015, participants received 22 e-questions divided into four categories: (1) Safety Policies, (2) Management’s Engagement, (3) Safety Programs and (4) Assessments of Safety Practices to determine retrospectively the training effectiveness of biosafety practices in their place of employment 6 months before and after attending their course. We used Kirkpatrick model to assess the transference of knowledge, skills and obstructive factors.

**Results:**

20% (81/402) of the participants completed the e-questionnaire. The overall percentage of positive responses indicating implementation of new safety practices increased from 50% to 84%. Improvement occurred in all four categories after attending the course, with the greatest increases in Safety Policies (67–94%) and Safety Programs (43–91%). Creating a safety committee, allocating resources, and establishing a facility safety policy were important drivers for implementing and maintaining laboratory safety practices. In addition, accredited laboratories and countries with national safety regulations or policies had a higher percentage of improvements. The most reported challenges were inadequate funding and lack of management enforcement.

**Conclusions:**

PEPFAR and other partners’ investments in training institutions, such as ACILT, were effective in building sustainable country ownership to strengthen biosafety practices and were leveraged to combat zoonotic diseases and COVID-19. Although support continues at the national/regional level, a standardized, coordinated and continent-wide sustainable approach to offer a biosafety program-like ACILT is missing. Continuous offerings of biosafety programs similar to ACILT could contribute to sustainable strengthening of laboratory biosafety, QMS and pandemic preparedness.

**Supplementary Information:**

The online version contains supplementary material available at 10.1186/s41182-023-00557-1.

## Introduction

Sub-Saharan Africa (SSA) continues to face increased burden of diseases, such as HIV, tuberculosis (TB), malaria [1], Ebola [2], and the zoonotic diseases [3], including COVID-19 [4] [5]. As a result, government authorities face challenges to existing public health systems infrastructure associated with laboratory systems, disease prevention and control, and patient care management [1, 6, 7].

To address the afore mentioned needs the U.S. Centers for Disease Control and Prevention (CDC), the U.S. President’s Emergency Plan for AIDS Relief (PEPFAR), and the South African National Health Laboratory Service (NHLS) launched African Center for Integrated Laboratory Training (ACILT). From 2007 to 2016, ACILT provided free, hands-on training courses for laboratorians with a goal to prepare a competent laboratory workforce, strengthen laboratory systems, enhance diagnostics capacity in SSA [8].

Long before COVID-19 pandemic, because of disease outbreaks such as Zika, Marburg, and Ebola viruses and the growing fear of bioterrorism, strengthening biosecurity and biosafety issues had become an urgent global goal [9]. ACILT’s biosafety program was designed to guide institutions to protect laboratory workers, the public, and the environment from potentially hazardous biological agents. The goal was to assist Ministry of Health’s (MoH) hospital and medical laboratories with Quality Management System (QMS) accreditation efforts, through strengthening laboratory biosafety and biosecurity practices. The program integrated 14 core laboratory safety elements in alignment with the requirements of ISO 15189 [10], ISO 15190 [11], ISO 45001 [12], ISO 35001 [13] and the WHO’s biosafety guidance with a focus on a risk-based approach [14].

ACILT’s laboratory biosafety program constituted of a 5-day Laboratory Biosafety and Infrastructure course offered 20 times between 2008 and 2014 to participants from 32 PEPFAR-supported countries. Biosafety was defined as a set of containment principles, technologies and practices that are implemented to prevent the unintentional exposure to biological agents or their inadvertent release [15]. In addition, biosecurity in ACILT’s program was defined as protecting biological agents from loss, theft, or misuse [16].

In 2015, e-questionnaires for all courses offered at ACILT, including biosafety, were sent to the 867 participants from 43 countries who attended between 2008 and 2014. A previously published manuscript [8] provides a holistic analysis of the combined transference of knowledge of 867 participants from 43 countries attending 75 course offerings [8]. This manuscript will describe the analysis from e-questionnaires sent to the 402/867 participants attending 20 biosafety courses from 32 countries. It summarizes the transference of knowledge, skills and abilities upon ACILT’s biosafety course completion and gauges enabling and inhibiting factors in implementation of biosafety programs, after participants return to their place of employment.

## Methods

### Study design

**Fig. 1:**
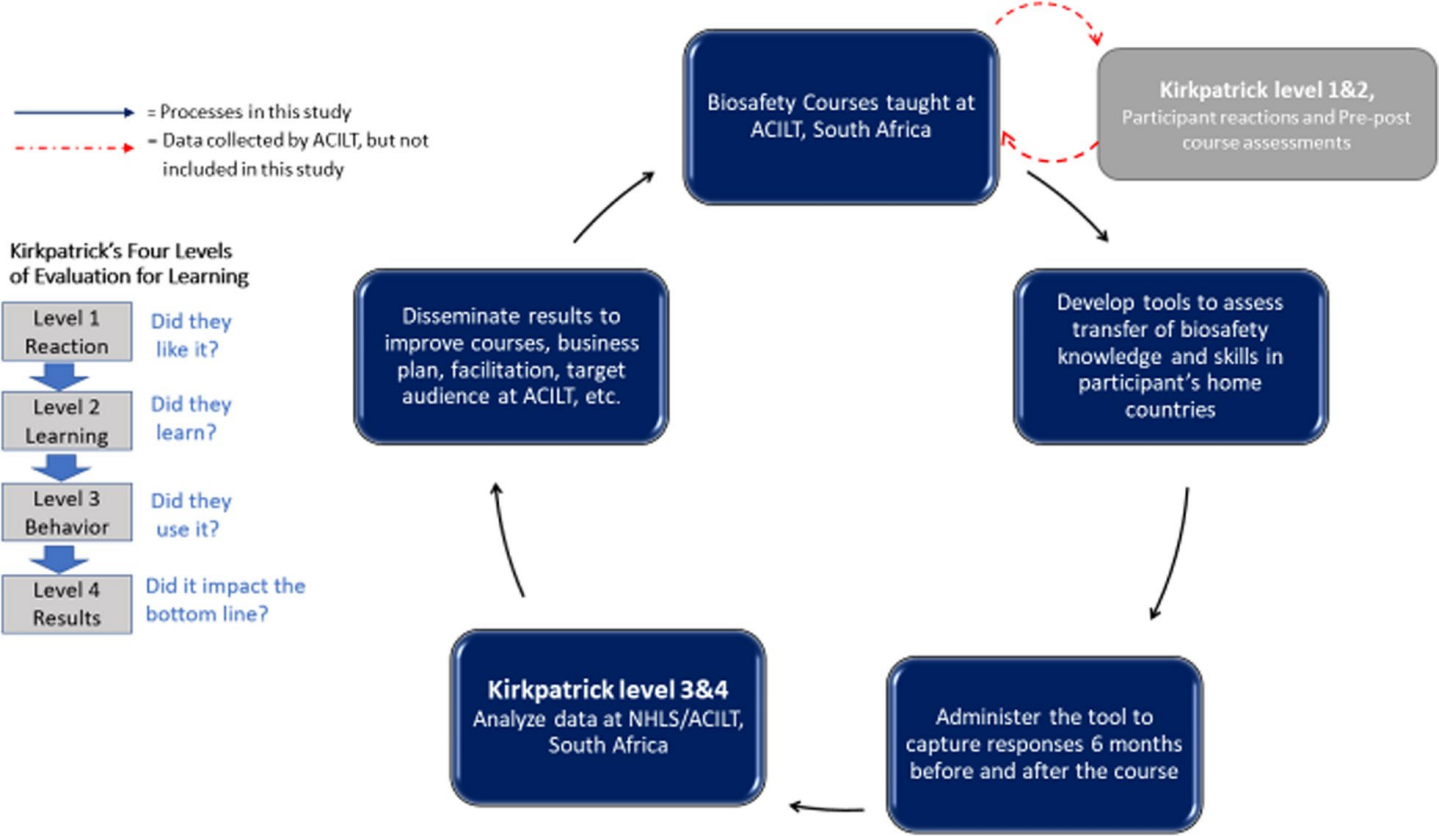
Workflow diagram to measure training effectiveness for ACILT’s Biosafety course using Kirkpatrick model. The blue boxes and arrows show the data that are reported in this study, whereas the grey box and red arrows show data that was collected at ACILT but not included in this study

A retrospective and cross-sectional study was designed using levels 3 and 4 of the Kirkpatrick models [17] to determine training effectiveness by assessing the transference of newly gained knowledge and skills to participant’s place of employment (Fig. 1). The following four levels were used to evaluate training using the Kirkpatrick model: [1] student’s reaction to the training experience; [2] increase in student’s knowledge from the training experience; [3] student’s self-reported behavioral change after applying the skills on the job and [4] student’s performance and impact on the business/organization. Levels 1 and 2 of the Kirkpatrick models were evaluated during the training and are not part of this study (Fig. 1).

### Training effectiveness

Defined as the extent to which course participants use their newly gained knowledge, skills and behaviors in their place of employment. "Behaviors” imply that the trainees successfully transferred the acquired knowledge, skills to others in their organizations which translated into biosafety program improvements.

### Course participants

Each of the 32 countries country selected and paid for their biosafety officers, laboratory managers, bench staff, professional trainers, institutional management, and facility engineers. For a more detailed breakdown of demographic characteristics of participants please refer to a previous publication [8].

### Inclusion/Exclusion criteria

Responders who completed the questionnaire and took the biosafety course at ACILT were included. Respondents who were not directly associated with a laboratory were excluded, e.g., program personnel, trainers, advisors etc.

### ACILT biosafety program development

The objectives of the program were designed to enable laboratory personnel and management to:

*Understand* the concepts of laboratory safety and introduce the 14 core safety elements; *Assess* the current safety programs, regulations considering accreditation requirements; *Develop* a tiered plan, budget and timeline to implement and/or strengthen safety elements; *Sensitize* and ensure necessary funds and resources; *Establish* routine review and assessments, for continual improvement and sustainability.

The course stressed the routine evaluation and implementation of 14 safety elements consolidated from global guidance for biological laboratories, as outlined in Table 1 [18–24]. Course developers prioritized, combined, and condensed several elements into modules as follows:

- Five elements prioritized laboratory accreditation: (#2—Safety Administration; #3—Hazard, Risk Assessment including biosafety levels; #5—Building and Facility Maintenance; #6—Safety Equipment and Maintenance; #11—Transport of Biological Agents).
- Seven elements combined laboratory safety operations: (#1—Management Responsibilities; #4—Occuptational Health; #7—Chemical Management, #8—Waste Management, #9—Emergency Preparedness; #10—Biosecurity; #12—Employee Training).
- Two elements combined laboratory hazards assessment: (#13—Radiation Safety; #14—Off-site activities).

**Table 1:** Description of 14 Core Laboratory Safety Elements taught at ACILT (2008–2014) in the Laboratory Biosafety and Infrastructure course

Core laboratory safety elements	Description
(1) Management’s Responsibilities*	Delegate, Resource, Implement, Enforce and Review
(2) Safety Business and Administration	Regulations, Policies, Committees, SOPs, Accreditations, IT-Systems, Communication-Public Relations
(3) Hazard/Risk Assessment Process	Review of Laboratories, Vivarium, and Areas that use/exposure to biological agents. (Hazard identification, Protection and Mitigation plans/processes)
(4) Occupational Health	Employee Medical Surveillance Program (Pre-exposure program, Emergency First-Aid training, Post-exposure protocol and Follow-up process.)
(5) Building/Facility and Maintenance	Building infrastructure, Safety-codes and systems (Mechanical/Ventilation, Electrical, Plumbing, Sewage, etc.)
(6) Safety Equipment and Maintenance	Personal Protective Equipment (PPE), Biosafety Cabinets, Small/Large-Safety equipment and instruments
(7) Chemical Management and Industrial Hygiene	Chemical safety program, use, accountability, and oversight (Cradle-to-grave responsibilities)
(8) Waste Management and Environmental Safety	Waste segregation, Labeling, Secure storage, Monitoring, Decontamination, Transport and Disposal methods (Safety oversight, responsibility accountability, and documentation)
(9) Emergency Preparedness and Response	Emergency Action Plan (Internal and External with local authorities), Communication Plan, Call-down Roster, and Drills
(10) Laboratory Biosecurity and High Hazard Disease Causing Agents	Delegation, Regulatory training, Proficiency, Implementation, Oversight and Documentation
(11) Transport of Biological Agents	Delegation, Regulatory training, Proficiency, Implementation, Oversight and Documentation
(12) Employee Training and Outreach	Hazard awareness, Protection mitigation processes, Practice demonstrations, Proficiency, Documentation
(13) Radiation Safety	Radiation compliance and permits, Radioisotopes, Laser safety, etc.
(14) Off-site satellite field and Laboratory Activities	Safety review of off-site activities involving use or exposure to hazardous biological agents

^*^Management must delegate oversight for each of the 14 core laboratory safety elements and ensure annual review. Laboratory Safety Officer (biosafety officer) must have a detailed understanding of how each of these 14 core laboratory safety elements and concepts support the safe operation of biological laboratories

Several of the elements required professionally trained experts to administer the programs, e.g., occupational health clinic, building engineering program, chemical and waste management programs, etc.; therefore, the course facilitators made two important recommendations: (a) the laboratory biosafety officer must have a detailed understanding of 14-elements. (b) Facility’s management must oversee and delegate responsibilities, ensure appropriate expertise and resources for annual review and compliance; identify strengths, weaknesses, and necessary recourses for continual improvement and sustainability of the safety efforts.

A pre and post-test was administered to all students at the beginning and end of the course (data not included in this study). The course was taught in a training-of-trainer (TOT) format and students were given electronic copies of the training materials, encouraged to adapt the materials to their local context and perform trainings upon return.

### Development of e-questionnaires

The evaluation e-questionnaire (Additional file 1) was developed in English with input from monitoring and evaluation advisors, course instructors and subject matter experts (SMEs) from the International Laboratory Branch (ILB) in the Division of Global HIV and TB at CDC Atlanta, Georgia, USA. It was designed to assess how well the afore-mentioned course objectives were accomplished while implementing in participant’s respective facilities. The questionnaire was structured into sections for I. Demographics, II. Transfer of Applied Skills and Knowledge, III. Change in Results and Processes, IV. Successes and Challenges and V. Recommendations. Sections III, IV and V had open ended questions. The questionnaire was piloted with laboratory professionals who did not participate in the training and consisted of 22 questions to assess safety practices 6 months before and after the course.

### Data collection

After obtaining voluntary consent to provide input to the e-questionnaires via online Survey Gizmo (https://forms.surveygizmo.com/plans-pricing/) or paper-based survey questionnaires for laboratories with poor internet connections, the final questionnaires were sent to the participants with a 2-week deadline for response. Study coordinators also sent two follow-up reminders at 14 and 21 days to non-responders. For those participants with poor internet connections, 35 resident CDC Laboratory Advisors were contacted to deliver questionnaires and then securely email the responses to the study coordinators who entered them into the database. Survey Gizmo only allowed each registered responder to submit one questionnaire. Access to Survey Gizmo was password protected.

### Data analysis

Participants were counted as responders if they returned the completed survey. An analysis (qualitative or quantitative) was performed on individual questions and was limited to the responders that completed a specific question.

### Qualitative data

With an aim to minimize bias in the analysis, the compiled data were examined independently by two study teams who created general categories. These categories were then divided into sub-categories to identify a relationship between them (coding). Following this approach, we captured key positive factors and challenges affecting the transfer of knowledge in the field. Some qualitative questions used a Likert scale from 1 to 5, where 1 is a very low response rate at 0%, 2 = 25%, 3 = 50%, 4 = 75% and 5 being the highest at 100%. For other qualitative responses, such as challenges and recommendations, analyzed responses were overlapping and not mutually exclusive.

### Quantitative data

To aggregate responses, the sum of affirmative responses was used as the numerator and the sum of responders who attempted the question was used as the denominator. A “not applicable” response to a question was included as a valid and affirmative response. For analysis, ACILT’s team shared de-identified data (personal identifiable information was not visible) with the study team at CDC headquarters in Atlanta, USA and CDC South Africa. The analyses were conducted with Statistical Analysis System v9.44 and Microsoft Excel based on questions divided into four categories (Table 2): (1) existing National Safety Policies, (2) management’s engagement in safety practices, (3) safety programs, and (4) performance of safety assessment following the course.

**Table 2:** Questions to evaluate the transference of knowledge and skills from ACILT’s Laboratory Biosafety and Infrastructure course to improve laboratory biosafety programs in participant’s respective laboratories in 22 countries (2008–2014) 6 months before and after the course

Part 1: Questions on safety policies	
Q4	Does the institution have ‘policies and guidance’ which indicates that management supports the implementation of laboratory safety programs?
Q5	Has the institution provided ‘resources’ (workplace, funding, staff, and materials), to indicate that management supports the implementation of laboratory safety programs?
Q6	Are laboratory safety strategies, goals, and objectives being developed and implemented?

Part 2: Questions on management’s engagement in safety practices	
Q 13	Has management provided appropriate staffing for implementation of the above safety program?
Q 14	Has management agreed to provide annual funding to implement the above safety programs and activities?
Q 15	Has management provided appropriate facilities and ancillary support to implement the above safety programs and activities?
Q 16	Has the institution and laboratories have developed a schedule process to re-evaluate safety and progress at defined intervals? (Ex. Quarterly, Semi-annual, Annual meetings or assessments)

Part 3: Questions pertaining to safety programs	
Q7	Does the institution/agency have a laboratory ‘Safety Committee,’ or ‘biosafety Committee’?
Q9B	As a result of the assessment, have new or existing programs been implemented and/or strengthened?
Q11	Has a strategy or plan(s) been developed to address and implement the above laboratory safety programs, as a result of the above evaluations?
Q17	Have new or existing biosafety programs increased compliance with: (a) local and national safety policies and regulations? (b) Laboratory accreditation efforts?

Part 4: Questions on performance of safety assessment	
9	Have the following laboratory safety program been evaluated and documented to identify gaps and potential hazards/risk to the employee, institution, and the environment?
9a	Laboratory 'Hazard Assessment" of activities and personnel involved in working with biological agents, also referred to as a 'biosafety risk assessment'?
9c	Review of Employee Occupational Health/Infection Control programs
9e.1	Assessment of Personal Protective Equipment (PPE)
9e.3	Assessment of safety equipment (Ex: biological safety cabinets, autoclaves, centrifuges, etc.)
9f	Assessment of Building and Facility safety
9j	Chemical Management program evaluation
9l	Waste Management program evaluation
9n	Laboratory ‘biosecurity’ evaluation
9t	Review of employee training programs

Serial number on the questions correspond to the original questionnaire available in the Additional file attachment

The training effectiveness was measured by the change in indicators collected 6 months before (baseline) and after the training at ACILT. Change in safety practices was captured with a with a required Yes/No response, and "Yes” was documented as a positive response. Changes were measured by improvements in safety practices at the participant’s facility using the absolute difference and change in percentage methods. The absolute difference was calculated by subtracting the Before ACILT’s course value (B) from the After value (A). The change in percentage or percent change was calculated by subtracting the B from A divided by B, (A–B/B). The following color scheme was used to show gradients in changes before or after the course: ≤ 25% dark red, 25–50% peach, 51–89% light green and ≥ 90% dark green.

## Results

**Fig. 2:**
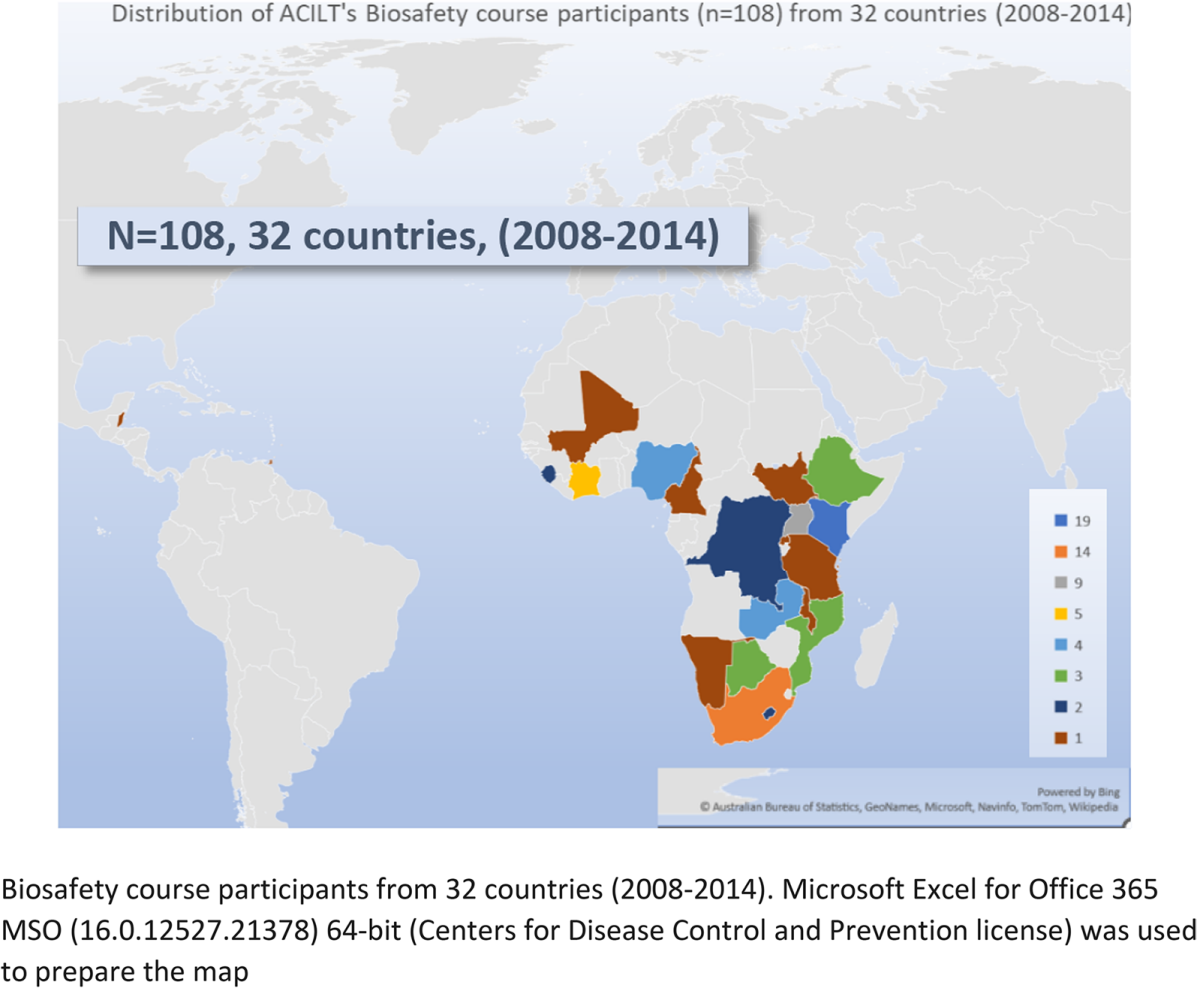
Global distribution of countries that participated in the biosafety training and numbers of participants. Colors in the legend depict the number of participants from that country

Total of 108/402 participants (Fig. 2) from 32 countries returned the survey. Based on the exclusion criteria 27 participants from 10 countries were excluded. The final response rate was 20% (81/402), from 22 countries—Barbados, Belize, Botswana, Cameroon, Cote d'Ivoire, Democratic Republic of Congo, Ethiopia, Kenya, Lesotho, Malawi, Mali, Mozambique, Namibia, Nigeria, Rwanda, Sierra Leone, South Africa, South Sudan, Tanzania, Trinidad and Tobago, Uganda, Zambia. The respondents’ positions included 70% (57/81) in laboratory management and 30% (24/81) were technical professionals. Of these respondents 83.5% (68/81) were aware of their country’s National Safety Laws and Policies, and 37% (30/81) belonged to an accredited laboratory. Only 1 participant changed jobs after taking the course.

**Fig. 3:**
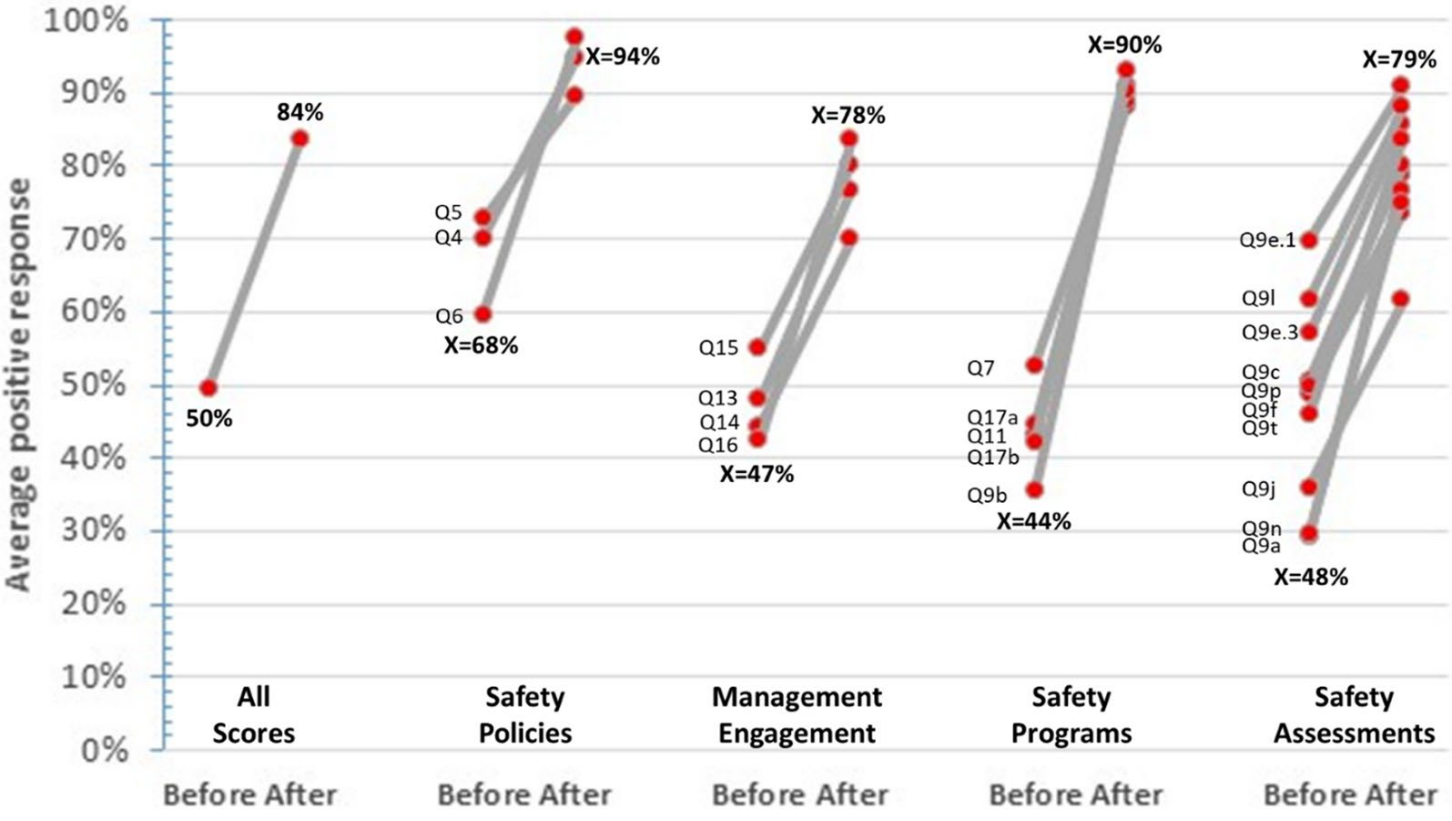
Change in average positive survey responses for participants (n=81) from 22 countries in respective laboratories following ACILT’s course (2008-2014) to implement biosafety practices

The overall percentage of safety practice positive responses improved from 50% before to 84% after the ACILT course as noted by all respondents to the survey (Fig. 3). The greatest increase in average positive responses was noted for Safety Programs (44–90%). All other safety areas had an increase in positive responses from before to after the course: Safety Policies (68–94%), Management Engagement (47–78%), and Assessments (48–79%).

**Fig. 4:**
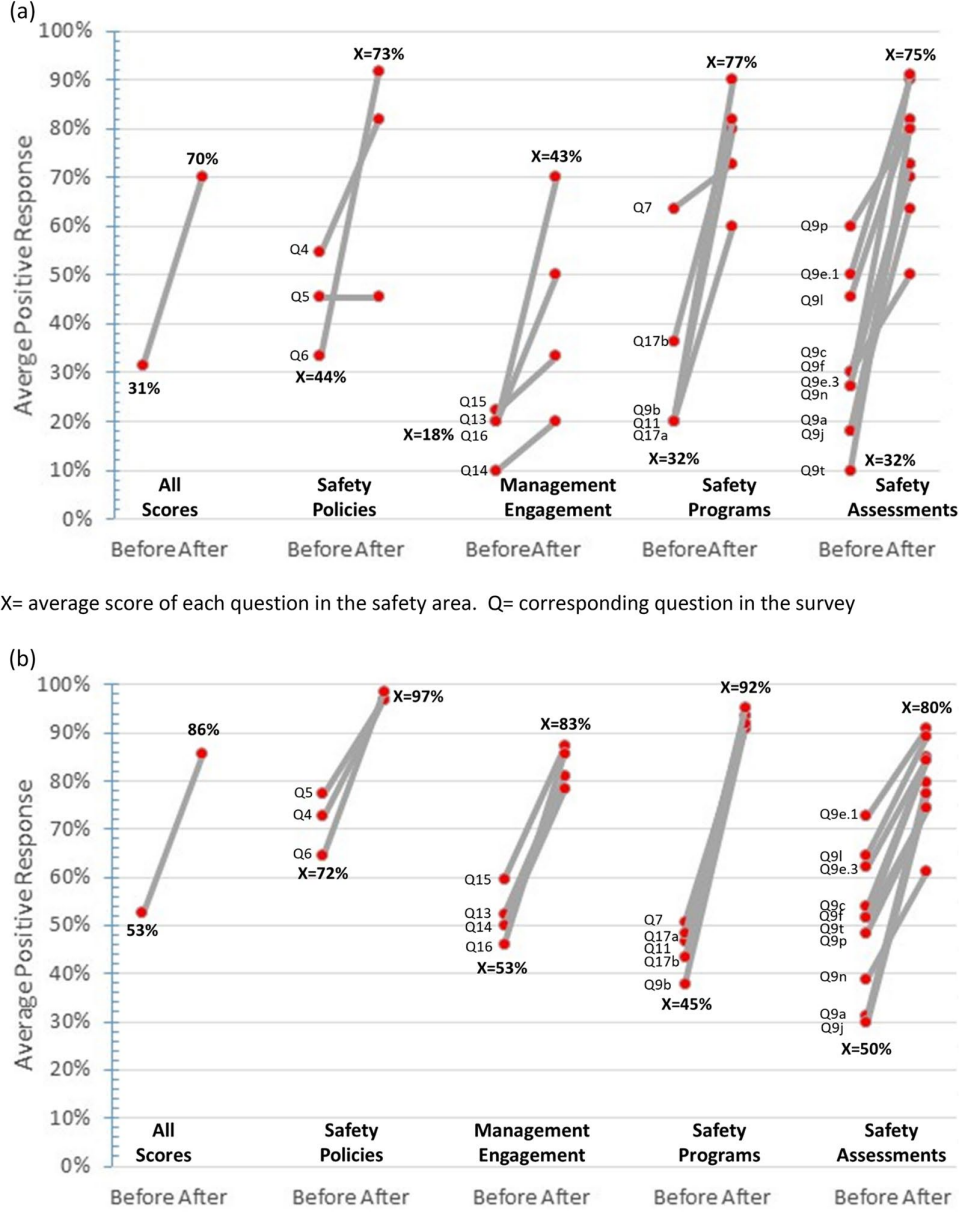
Effect of awareness for National Safety Policies on average positive responses from 22 countries following ACILT’s biosafety course (2008-2014). **a** Participants unaware of National Safety Regulations or Policies (n=13) **b** Participants aware of National Safety Regulations or Policies (n=68)

Respondents who were unaware (*n* = 13) if their country had a National Safety Regulations or Policy, indicated only 31% overall positive responses to safety practices prior to the course. (Fig. 4a). While those respondents who were aware (*n* = 68) of their country’s National Safety Regulations or Policy had 53% positive responses to the survey’s question prior to the ACILT’s course (Fig. 4b). Both respondents who were unaware or aware of their National Safety Regulations or Policy had higher positive responses to the survey after the ACILT course, increasing to 70% and 86%, respectively. Of note, those respondents unaware of their national policies or regulations had low positive responses for the management engagement question both before (18%) and after (43%) the course.

The results of the survey indicated how a laboratory’s status before the course regarding accreditation, safety policies, safety strategies and goals, and resources for safety influenced the implementation of biosafety practices in participant’s laboratories (Table 3).

i. Laboratory Accreditation: Prior to the course, respondents from accredited laboratories had a higher initial percentage of positive responses (67%) for safety practices when compared to non-accredited laboratories (40%). After the course, safety practices positive responses increased to 93% in accredited laboratories and 78% in non-accredited laboratories.
ii. Facility Safety Policy: Respondents from laboratories without a safety policy prior to the course but having developed one after the course showed increased positive responses to safety practices from 14% to 78%. Respondents whose facilities never (before or after the course) had safety policies in place had a modest increase in favorable response from 11% to 48%. Those respondents with a safety policy prior to the course had a highest percentage (65%) of positive response which increased further after the course (88%).
iii. Laboratory Strategies: Respondents from laboratories that had laboratory safety strategies and goals (*n* = 46) had a higher positive response to the survey question prior to the course (68%) when compared to laboratories (*n* = 30) without (24%). Following the course, both respondent groups’ positive response increased to 86% and 83%, respectively.
iv. Facility Safety Resources: Respondents from laboratories without sufficient resources prior to the course but allocated after the course, showed increased positive responses to the survey from 15% to 86%. Respondents whose facilities never had allocated resources had a modest increase in positive response from 13% to 53%. Respondents indicating availability of resources prior to the course had the highest percentage (63%) of positive response which further increased after the course (87%).

**Table 3:**
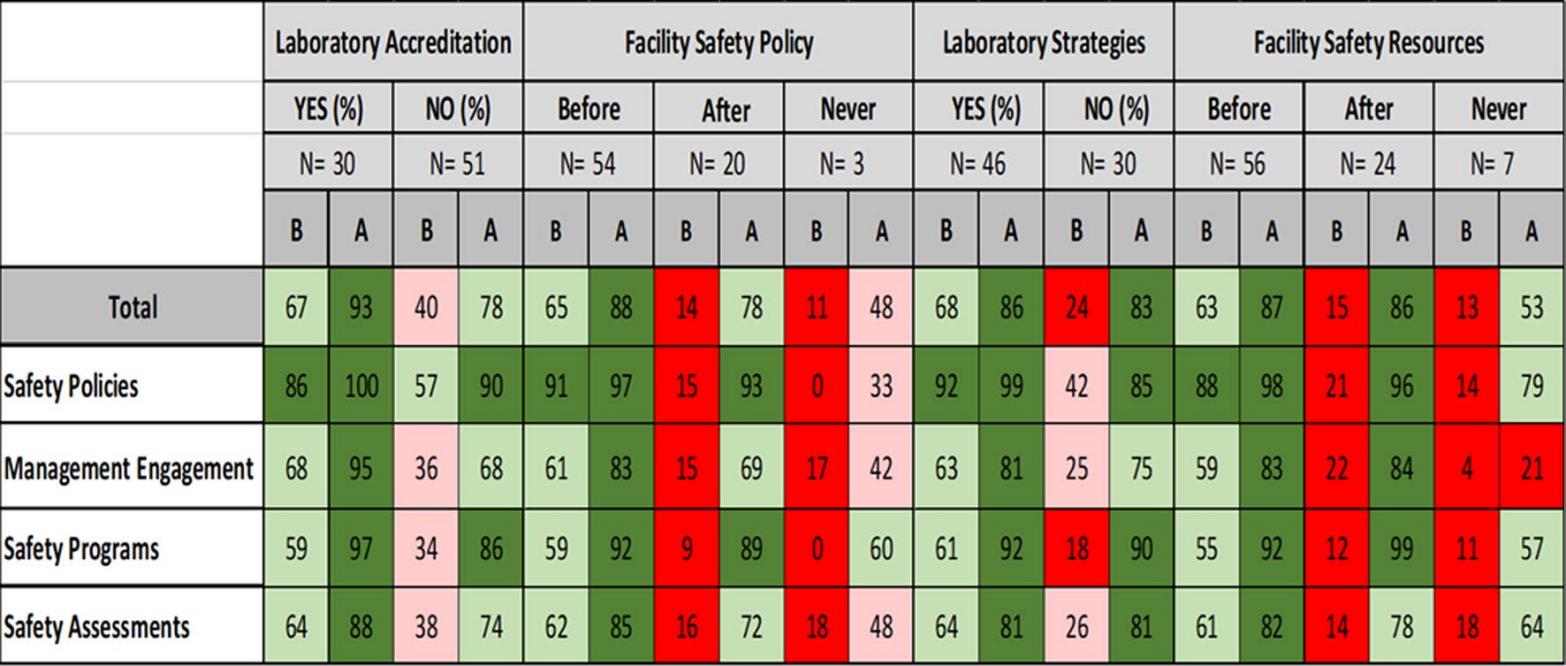
Impact of laboratory accreditation, facility safety policies, laboratory safety strategies and facility resources on implementation of biosafety practices in the participant’s laboratory before and after the ACILT course (2008–2014)

Color coding: ≤ 25% = Dark Red; (26–50%) = Peach; (51–79%) = Light green; ≥ 80 = Dark green

^*^B = Before participating in ACILT course

^**^A = After participating in ACILT course

The results of the survey indicated that laboratory personnel that have a safety committee or biosafety officer influenced the implementation of biosafety practices in participant’s laboratories (Table 4).

**Table 4:**
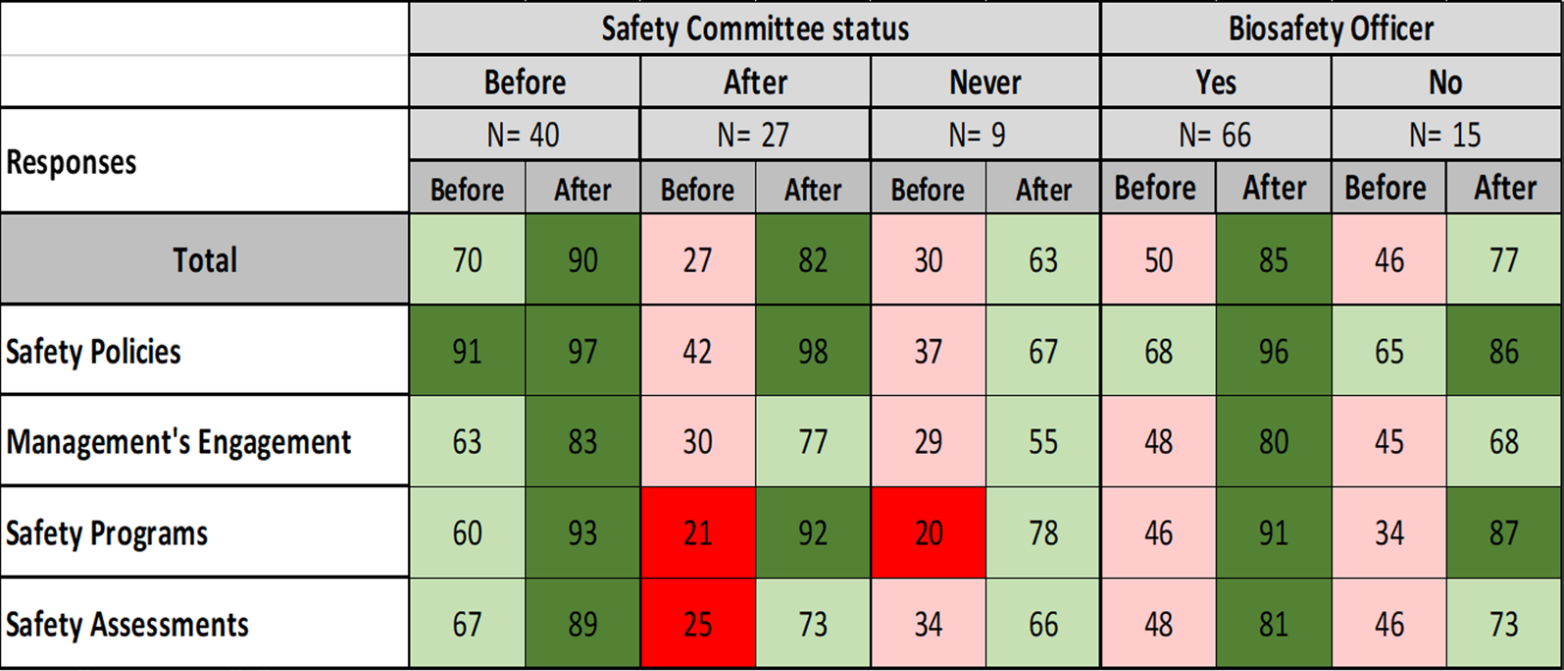
Impact of Facility Safety Committee status and having a biosafety officer on implementation of biosafety practices in the laboratory before and after the ACILT course

Color coding: ≤ 25% = Dark Red; (26–50%) = Peach; (51–79%) = Light green; ≥ 80 = Dark green

i. Laboratories having a safety committee resulted in higher positive responses to ACILT’s safety survey than laboratories without a safety committee. Respondents (*n* = 27) from laboratories without a safety committee prior to course but formed one after the course increased positive responses to the survey from 27% to 82%. Respondents (*n* = 9) whose laboratory never had a safety committee had a modest increase in positive responses from 30% to 63%. Of note those respondents (*n* = 40) with a safety committee prior to the course had a higher percentage (70%) of positive responses which increased after the course (90%).
ii. Having a biosafety officer in the respondent’s laboratory (*n* = 66) had a similar percentage of positive responses to laboratories without a biosafety officer (*n* = 15) prior to, 50% and 46%, and after, 85% and 77%, the course, respectively.

Participants provided answers about challenges and barriers to implementing newly learned safety practices after the course (Table 5). The most reported challenge was with funding (*n*=40), followed by lack of management support (*n*=17) and country-specific delays (*n*=2), e.g., lack of an institutional committee to deal with safety program.

**Table 5:** Collated responses for challenges and improvements to ACILT’s biosafety course (2008–2014)

*Qualitative analysis of responses for Biosafety Course	
Challenges and Barriers	1. Funding: (n=40) a. No budget for safety programs b. Difficulty accessing funds c. Limited funding d. Austerity measures 2. Lack of Compliance or Support from Management: (*n* = 17) a. Includes lack of support from government b. Convincing management to allocate funds for biosafety c. Low priority for management d. Bureaucratic issues e. Management does not make essential resources available to all employees 3. Country-specific delays: (*n* = 2) a. Delays in obtaining the permit by **DOH: b. There is no specific institutional committee to deal with the safety programs:
Suggested improvements	1. Extend the duration of and add refresher courses (*n* = 15) 2. More practical training: (*n* = 17) a. Have on-job training b. Hands-on practice 3. Course Design/Structure: (*n* = 19) a. Include scenarios from resource-limited countries b. Train the trainers (TOTs) c. Upgrading with changing times

*Responses are overlapping and not mutually exclusive ** DOH=Department of Health

Challenges and Barriers represent section IV and Suggested improvement represent section V of the questionnaire

3% (3/96) participants responded on a Likert scale of 1–5, (1 being 0% and 5 being the highest at 100%) that resources were always fully accessible to them; 56% (45/96) responded that resources were available between 50% and 75% of the time; 41% (41/96) belonging to mostly non-accredited laboratories, responded that resources were either not made available or were made available 25% of the time.

85% (82/96) of participants indicated on a Likert scale of 1–5, (1 being very low, and 5 being very high), that their motivation level ranged from high to very high to apply learning from the course in their own facility. Of the 40 highest motivated participants 25 belonged to laboratories that were not accredited and 13 came from accredited labs.

## Discussion

We found evidence for training effectiveness that the course familiarized participants on the core elements of laboratory safety and taught them how to successfully transfer learned knowledge and skills to their facilities. PEPFAR funded biosafety program at ACILT had an impact in building capacity for biosafety practices in participant’s laboratories. A systematic review based on 22 studies for hazards in occupational health and safety (OHS) programs traced evidence for training effectiveness, including (i) knowledge, (ii) attitudes and beliefs, (iii) behaviors, and (iv) health outcomes. Strong evidence was found for the effectiveness of training on worker OHS behaviors, but insufficient evidence was found of its effectiveness on health outcomes [25]. Our study may be one of the few published on biosafety that have shown evidence for successful training effectiveness at levels 3 and 4 of Kirkpatrick model.

A higher number of participants from non-accredited laboratories were motivated to apply learning at their facilities after completing the course, which is indicative of a participant’s understanding and recognition of the importance of biosafety practices to achieve accreditation.

Our analysis identified three factors that shaped the response in strengthening biosafety programs at the participant’s facilities, both at facility and national level. These factors included having (a) a safety policy, (b) laboratory safety committee, and (c) dedicated available resources for safety. Furthermore, countries with accredited laboratories, national safety regulations, laboratory strategies and goals, and designated biosafety officers showed improvements in biosafety programs. Our findings are corroborated by studies providing suggestions for sustainable capacity development for biosafety and biosecurity challenges in low resource countries [26], important role of biosafety officers [27] and lapses in laboratory biosafety operations due to lack of one [28].

Our results also confirmed that facility management plays a key role in executing and sustaining safe laboratory operations to provide the necessary resources and ensure implementation of biosafety and biosecurity programs. A strong management commitment showed positive impact on biosafety and biosecurity aspects in Indonesian laboratories too [29].

Improvements in biosafety practices lead to strengthened laboratory operations, preparedness for accreditation, responses to public health emergencies and outbreaks and systems for sustained HIV and TB epidemic control [30, 31]. Between 2017 and 2022, the number of PEPFAR-supported facilities with a molecular laboratory increased by 115%, from 926 to 1,995; and those that were accredited increased by 194%, from 103 to 303 [32]. This enormous growth in accreditation could not be possible without competent biosafety personnel in each facility. Uganda and Kenya with 53 and 85 accredited laboratories, respectively (December 2022), are shining examples of sustainability of biosafety program [33, 34].

Apart from PEPFAR there are additional external factors that have contributed to the successful implementation of the biosafety and biosecurity programs in countries, such as: international policies, e.g., WHO’s International Health Regulations [35]; programs, e.g., Global Health Security Agenda [36] and Global Fund to Fight AIDS, Tuberculosis and Malaria (GF) [37]; partners, e.g., PEPFAR’s Public–Private Partnership [38]; and declarations, e.g., Maputo Declaration [39]. Case in point, in 2007 Kenya received funding from the GF and World Bank (WB) to develop a policy on waste management and a training model to strengthen biosafety and biosecurity laboratory systems [40]. In 2011, WB funded laboratory accreditation efforts, including funds for biosafety, in six laboratories in Kenya and other countries such as Uganda, Tanzania, and Rwanda through the East Africa Public Health Laboratory Network initiative.

### Leveraging ACILT’s biosafety program for COVID-19 and other zoonotic diseases—country examples

“ACILT’s biosafety program aimed to reduce risk of occupational and community spread of HIV and TB but had collateral benefits extending to multiple healthcare-associated communicable diseases, such as COVID-19, Ebola, hepatitis and others”—(Communication from Dr. Jane Mwangi, CDC, Kenya).

Following CDC Kenya’s financial support to attend ACILT’s biosafety program, the MoH elevated the health and safety section of the nursing department to a stand-alone, fully staffed Infection Prevention and Control (IPC) Unit. A biosafety unit was established at National Public Health Laboratory Services (NPHL). This unit established Training-of-Trainers to support annual biosafety refresher trainings. In 2019, during the COVID-19 pandemic, the MoH’s headquarter IPC Unit and the NPHL biosafety Unit developed online training material to enable health care workers safely collect, process and test samples safely, as well as handle bodies of deceased from COVID-19.

Between 2017 and 2018, Uganda experience eight disease outbreaks, including those from zoonotic diseases [41]. The outbreaks posed a high risk to the laboratory personnel involved in the outbreaks and response activities and required appropriate biosafety practices to prevent or reduce any exposures to infectious agents. Biosafety trainees both from MoH and partners’ institutions used the knowledge gained at ACILT to ensure safe sample collection, testing and waste management as they were part of a pool of trainers used by the MoH for most of the laboratory capacity building activities. (Communication from Mr. Joel Peter Opio, CDC Uganda).

ACILT’s biosafety program contributed to building sustainable biosafety capacity, long after it was offered. Like Kenya and Uganda many other countries utilized pre-existing healthcare infrastructure which proved to be an important asset in mounting an effective response against a health threat-like COVID-19 [42].

Throughout the COVID-19 pandemic, there was an extreme shortage of personal protective equipment, disinfectants, supplies, but the most extreme shortage was a deficit of qualified, trained staff, including biosafety personnel [43]. Therefore, to increase readiness for the next pandemic, funding and commitment from the governmental bodies to adapt biosafety and biosecurity policies in resource limited conditions were identified as a major need [44].

### Challenges

Challenges for funding and lack of management’s support are similar to what other experts have reported as keys to success for biosafety programs and include the importance of policies/strategic laboratory planning [45], strong multisectoral approach [46] and lack of financial resources [47].

### Limitations

Limitations to this study included low response rate (20%) partially due to limited access to internet in SSA countries, which was compounded by the mass transition of email addresses by PEPFAR programs from individual country emails to a common CDC email in 2015. A meta-analysis of 39 studies showed that web survey modes have on average a 10% lower response rates than mail surveys [48]. In another self-reported web survey study the overall non-response rate was higher in the self-administered mode (37.9%) than in the face-to-face interview mode (23.7%) [49]. Considering these studies, the response rate in this study is modest. Even with a modest response rate it is evident that participants were able to transfer knowledge and skills in their facilities. Second, the data were retrospectively collected, no statistical tests were conducted to ascertain the statistical significance for before and after the course responses. The data were also self-reported, which could be subject to social desirability, personal and recall biases. Finally, responses from e-questionnaires were de-identified so substantiation of the information, for responders and non-responders, irrespective of the duration of study, could have taken place by one, two or all three of ongoing acceptable systems at ILB a) online routine guidance communication offered by SMEs b) technical assistance visits to countries c) other PEPFAR program assessment reports [49].

## Conclusion

PEPFAR and other partner’s investments in training institutions, such as ACILT, were effective in building sustainable country ownership to strengthen biosafety practices and were leveraged to combat zoonotic diseases and COVID-19. While support continues at the national/regional level, however, a standardized, coordinated and continent-wide sustainable approach to offer a biosafety program-like ACILT is missing. Continuous offerings of biosafety programs similar to ACILT could contribute to sustainable strengthening of laboratory biosafety, QMS and pandemic preparedness.

### Supplementary Information

**Additional file 1.** Laboratory Biosafety & Infrastructure Course ACILT Program Evaluation Questionnaire.

## Data Availability

The data sets used and/or analyzed during the current study are available from the corresponding author on reasonable request.

## Abbreviations

ACILT: The African Center for Integrated Laboratory Training
PEPFAR: The U.S President’s Emergency Plan for AIDS Relief
SSA: Sub-Saharan Africa
TB: Tuberculosis
CDC: U.S. Centers for Disease Control and Prevention
NHLS: National Health Laboratory Service in South Africa
MoH: Ministry of Health
QMS: Quality Management System
SME: Subject matter expert
ILB: International Laboratory Branch
OHS: Occupational Health and Safety
GF: Global Fund to Fight AIDS, Tuberculosis and Malaria
IPC: Infection Prevention and Control
NPHL: National Public Health Laboratory Services

## References

[1] Nkengasong JN (2009). Strengthening laboratory services and systems in resource-poor countries. Am J Clinical Pathol..

[2] Sealy TK, Erickson BR, Taboy CH, Stroher U, Towner JS, Andrews SE (2016). Laboratory response to Ebola—West Africa and United States. MMWR Suppl.

[3] Buregyeya E, Atusingwize E, Nsamba P, Musoke D, Naigaga I, Kabasa JD (2020). Operationalizing the one health approach in Uganda: challenges and opportunities. J Epidemiol Glob Health.

[4] Nkengasong JN (2020). Let Africa into the market for COVID-19 diagnostics. Nature.

[5] Elton L, Haider N, Kock R, Thomason MJ, Tembo J, Arruda LB (2021). Zoonotic disease preparedness in sub-Saharan African countries. One Health Outlook.

[6] Alemnji G, Fonjungo P, Van Der Pol B, Peter T, Kantor R, Nkengasong J (2014). The centrality of laboratory services in the HIV treatment and prevention cascade: the need for effective linkages and referrals in resource-limited settings. AIDS Patient Care STDS.

[7] Birx DL, de Souza M, Nkengasong JN (2009). Laboratory challenges in the scaling up of HIV, TB, and malaria programs: the interaction of health and laboratory systems, clinical research, and service delivery. Am J Clin Pathol.

[8] Shrivastava R, Poxon R, Rottinghaus E, Essop L, Sanon V, Chipeta Z (2021). Leveraging gains from African Center for integrated laboratory training to combat HIV epidemic in sub-Saharan Africa. BMC Health Serv Res.

[9] Henry M, Ogaro CK, Mbatha S, Ngayo MO (2018). Biorisk status: a comparative assessment of private and public medical diagnostic laboratories in Western Kenya. Appl Biosafety.

[10] International Organization for Standardization. ISO 15189:2012 Medical Laboratories—Requirements for Quality and Competence, Geneva, 2012 [https://www.iso.org/obp/ui/#iso:std:iso:15189:ed-3:v2:en 10.1111/j.1423-0410.2002.tb05329.x 12617164

[11] International Organization for Standardization. ISO 15190:2020(en) Medical laboratories—Requirements for safety 2020. https://www.iso.org/obp/ui/#iso:std:iso:15190:ed-2:v1:en. Accessed June 2021.

[12] International Organization for Standardization (ISO). ISO 45001: 2018 Occupational health and safety management systems—Requirements with guidance for use 2018.

[13] International Organization for Standardization (ISO). ISO 35001:2019 Biorisk management for laboratories and other related organisations. 2019.

[14] World Health Organization. WHO GUIDANCE on implementing regulatory requirements for biosafety and biosecurity in biomedical laboratories–a stepwise approach 2020. https://apps.who.int/iris/handle/10665/332244.

[15] World Health Organization. Laboratory Biosafety Manual 2004 [3rd:[181]. https://www.who.int/publications/i/item/9241546506.

[16] World Health Organization. Biorisk management : laboratory biosecurity guidance 2006 [https://apps.who.int/iris/handle/10665/69390.

[17] Kirkpatrick J, Kirkpatrick WK. The Kirkpatrick Four Levels™: A Fresh Look After 50 Years 1959 - 2009 2009 [3]. https://openspaceconsulting.com/wp-content/uploads/2019/06/Kirkpatrick-Four-Levels-wp-updated.pdf.

[18] World Health Organization. ISO 15189:2012(en) Medical laboratories — Requirements for quality and competence 2012 [https://www.iso.org/obp/ui/#iso:std:iso:15189:ed-3:v2:en.

[19] World Health Organization. ISO 15190:2020 Medical laboratories—Requirements for safety (ISO - ISO 15190:2020 - Medical laboratories—Requirements for safety). 2020.

[20] World Health Organization. Laboratory Biosafety Manual. Third ed2004. 24404640

[21] Diane O. Fleming, Debra L. Hunt. Biological Safety: Principles and Practices. 4th ed: ASM Press; 2006.

[22] Stevens T. CDC’s Development of a Biosafety Assessment Tool. In: News for the Federal Biorisk Management Policy Community HHS, editor.: Janelle Hurwitz; 2013.

[23] International Organization for Standardization. CEN Workshop Agreement 15793:2011, Laboratory Biorisk Management 2011.

[24] International Organization for Standardization. CEN Workshop Agreement 16393:2012, Laboratory Biorisk Management-Guidelines for the implementation of CWA 15793:2008 2008

[25] Robson LS, Stephenson CM, Schulte PA, Amick BC, Irvin EL, Eggerth DE (2012). A systematic review of the effectiveness of occupational health and safety training. Scand J Work Environ Health.

[26] Heckert R, Reed C, Gmuender F, Ellis M, Tonui W (2011). International biosafety and biosecurity challenges: suggestions for developing sustainable capacity in low-resource countries. Applied Biosafety.

[27] Kaufman SG, Mathews H, Alderman LM (2007). Biosafety officers, behavioral compliance strategies, and their effects on laboratory practices. Appl Biosafety.

[28] Oladeinde BH, Omoregie R, Odia I, Osakue EO, Imade OS (2013). Biorisk assessment of medical diagnostic laboratories in Nigeria. Saf Health Work.

[29] Bowolaksono A, Lestari F, Satyawardhani SA, Kadir A, Maharani CF, Paramitasari D (2021). Analysis of bio-risk management system implementation in Indonesian higher education laboratory. Int J Environ Res Public Health.

[30] World Health Organization. Public health emergencies: preparedness and response International Health Regulations (2005), Annual report on the implementation of the International Health Regulations (2005). 2019. Report No.: A72/8.

[31] World Health Assembly. Fifty Eight World Health Assembly: WHA58.29 Enhancement of laboratory biosafety. 2005.

[32] Chun HM, Dirlikov E, Cox MH, Sherlock MW, Obeng-Aduasare Y, Sato K (2023). Vital signs: progress toward eliminating HIV as a global public health threat through scale-up of antiretroviral therapy and health system strengthening supported by the U.S. President's emergency plan for AIDS relief—worldwide, 2004–2022. MMWR Morb Mortal Wkly Rep.

[33] Kyeyune Ali. DR JULIUS: “Public Should Be Critical Of Choice Of Laboratory Service Providers” 2022 [https://voiceofbugerere.com/12238-2/.

[34] (CDC). USPsEPfARPttUSCfDCaP. SLMTA Laboratories that have achieved accreditation: U.S. President’s Emergency Plan for AIDS Relief (PEPFAR) through the U.S. Centers for Disease Control and Prevention (CDC). [https://slmta.org/accredited-labs/.

[35] World Health Organization. International Health Regulations 2005 [https://www.who.int/health-topics/international-health-regulations#tab=tab_1

[36] Global Health Security Agenda. The 6th GHSA Ministerial meeting 2020 [cited 2021 November]. https://ghsagenda.org/

[37] U.S. President’s Emergency Plan for AIDS Relief. PEPFAR 2020 Annual Report to Congress. 2020.

[38] Shrivastava R, Gadde R, Nkengasong JN (2016). Importance of public-private partnerships: strengthening laboratory medicine systems and clinical practice in Africa. J Infect Dis.

[39] World Health Organization (2008). The Maputo declaration on strengthening of laboratory systems.

[40] Muriithi B, Bundi M, Galata A, Miringu G, Wandera E, Kathiiko C (2018). Biosafety and biosecurity capacity building: insights from implementation of the NUITM-KEMRI biosafety training model. Trop Med Health.

[41] Mbonye AK, Sekamatte M (2018). Disease outbreaks and reporting in Uganda. The Lancet.

[42] Romano ER, Sleeman K, Hall-Eidson P, Zeh C, Bhairavabhotla R, Zhang G (2022). Contribution of PEPFAR-Supported HIV and TB molecular diagnostic networks to COVID-19 testing preparedness in 16 countries. Emerg Infect Dis.

[43] Gillum DR, Rice AD, Mendoza IA (2022). The COVID-19 pandemic response: biosafety perspectives from a large research and teaching institution. Appl Biosafety.

[44] Rutjes SA, Vennis IM, Wagner E, Maisaia V, Peintner L (2023). Biosafety and biosecurity challenges during the COVID-19 pandemic and beyond. Front Bioeng Biotechnol.

[45] Ondoa P, van der Broek A, Jansen C, de Bruijn H, Schultsz C (2017). National laboratory policies and plans in sub-Saharan African countries: gaps and opportunities. Afr J Lab Med.

[46] Yeh KB, Adams M, Stamper PD, Dasgupta D, Hewson R, Buck CD (2016). National laboratory planning: developing sustainable biocontainment laboratories in limited resource areas. Health Secur.

[47] Rutebemberwa E, Aku FY, Zein E, Bellali H (2020). Reasons for and barriers to biosafety and biosecurity training in health-related organizations in Africa, Middle East and Central Asia: findings from GIBACHT training needs assessments 2018–2019. Pan Afr Med J.

[48] Shih T-H, Xitao F (2008). Comparing response rates from web and mail surveys: a meta-analysis. Field Methods.

[49] Christensen AI, Ekholm O, Glu¨ mer C, Juel K. (2013). Effect of survey mode on response patterns: comparison of face-to-face and self-administered modes in health surveys. Eur J Public Health.

