# Supplementary material for: Measuring training effectiveness of laboratory biosafety program offered at African Center for Integrated Laboratory Training in 22 President’s Emergency Plan for AIDS Relief supported countries (2008–2014)
Source: Trop Med Health. 2023 Nov 21;51:65. doi: 10.1186/s41182-023-00557-1 (PMC10662895; doi:10.1186/s41182-023-00557-1)
Supplement: Supplementary file 1 — Additional file 1. Laboratory Biosafety & Infrastructure Course ACILT Program Evaluation Questionnaire. [file 41182_2023_557_MOESM1_ESM.doc]

**Laboratory Biosafety & Infrastructure Course**

**ACILT Program Evaluation Questionnaire**

1. **Demographics – please give CURRENT information**

| Name (surname, given name): | |  | | | | | Age: | | |  | | Gender (circle one): | | M F | |
| --- | --- | --- | --- | --- | --- | --- | --- | --- | --- | --- | --- | --- | --- | --- | --- |
| Your institution name: | |  | | | | | Country name: | | | | |  | |  | |
| Your laboratory type (select one): | | O Reference O Hospital O Private O Non-Government Organization | | | | | | | | | | | | | |
|  |  | O Other, please specify: | | |  | | | | | | | | | | |
| Your highest education level (select one): | | | O Primary O Secondary O Certificate  O College Degree O Post-College | | | | | | | | | | | | |
|  |  |  | O Other, please specify: | | | | |  | | | | | | | |
| Your position (select one): |  | | O Safety Officer O Quality Management Officer  O Lab Supervisor/Manager O Other, please specify: | | | | | | | | | | | |  |
| Years in your position |  | | Years of laboratory experience: | | | | | |  | | Years of HIV lab experience: | | | |  |
| Are you still in the same job as when you took the course? | - Yes - No | | If no, please provide reason: | | | | | |  | | | | | | |
| Has your laboratory become accredited by an external organization? | - Yes - No | | If yes, what year: |  | | Who was the accrediting body? | | | | | | |  | | |
|  | If not, provide reason: | | |  | | | | | | | | | | | |

**Course specific Information**

| Course Location: |  | | Dates attended: |  |
| --- | --- | --- | --- | --- |
| Course name: | |  | | |

**II. Transfer of Applied Skills and Knowledge**:

Please select your response to the questions below and provide comments in the space available.

| **Question** | | **Answer** | |
| --- | --- | --- | --- |
|  | **Management’s Education & Responsibilities** |  |  |
| 1. | Does your country have national “workplace safety” regulations or laws that require employers to provide a safe workplace for employees? | Yes | No |
| 2. | Following the course did you **hold a debriefing for stakeholders** to share course concepts, importance, and how to implement ‘Laboratory Safety’? If yes, whom did you brief? | Yes | No |
| 3. | Laboratory Safety Officer | Yes | No |
|  | Laboratory Quality Management Officer | Yes | No |
|  | Laboratory Manager/ Supervisor/ Directorate | Yes | No |
|  | Institution / Agency-Level Management | Yes | No |
|  | Ministry Level Management | Yes | No |
|  | Funding Partners: PEPFAR or Global Fund (please circle) | Yes | No |
|  | Following the debriefing did the following stakeholders **acknowledge the importance** of laboratory safety and approve plan for program implementation? |  |  |
|  | Laboratory Safety Officer | Yes | No |
|  | Laboratory Quality Management Officer | Yes | No |
|  | Laboratory Manager/ Supervisor/ Directorate | Yes | No |
|  | Institution / Agency-Level Management | Yes | No |
|  | Ministry Level Management | Yes | No |
|  | Funding Partners: PEPFAR or Global Fund (please circle) | Yes | No |
|  | Other(s) (specify)__________________ | Yes | No |
|  | **Please evaluate the following results before and after training.** | **Before** | **After** |
| 4. | Does the institution have ‘**policies and guidance**’ which indicates that management supports the implementation of laboratory safety programs?  If no afterwards, then specify why : ________________________________________________________________________ | Yes/No | Yes/No |
| 5. | Has the institution provided **’resources’ (workplace, funding, staff, & materials)**, to indicate that management supports the implementation of laboratory safety programs?  If no afterwards, then specify why : ________________________________________________________________________ | Yes/No | Yes/No |
| 6. | Are laboratory safety **strategies, goals, and objectives** being developed and implemented?  If no afterwards, then specify why : ________________________________________________________________________ | Yes/No | Yes/No |
| 7. | Does the institution/agency have a laboratory ‘Safety Committee,’ or Biosafety Committee?  If no afterwards, then specify why: ________________________________________________________________________ | Yes/No | Yes/No |
| 8. | Do the following individuals participate in your institution’s safety or biosafety committee? |  |  |
|  | - Institution senior managing office | Yes/No | Yes/No |
|  | - Biosafety Officer / Safety Officer | Yes/No | Yes/No |
|  | - Laboratory Scientist (i.e., Principle Investigator) | Yes/No | Yes/No |
|  | - Medical Personnel (Employee Occupational Health / Infection Control Provider) | Yes/No | Yes/No |
|  | - Facility Manager/Engineer | Yes/No | Yes/No |
|  | - Security Officer | Yes/No | Yes/No |
|  | - Veterinary Officer / Animal Resource Manager (as appropriate) | Yes/No | Yes/No |
| **9. Program Evaluation:**  Have the following **laboratory safety programs been ‘evaluated & documented’** to identify gaps and potential hazards/risk to the employee, institution, and the environment? | | | |
|  | **A. Laboratory ‘Hazard Assessment”** of activities and personnel involved in working with biological agents, also referred to as a **‘Biosafety Risk Assessment’?**   - If no, then pick one: Lack of expertise; Funding; Time constraints; Not a management priority; Other:_____________________________________________________   **B.** As a result of the assessment, have new or existing programs been implemented and/or strengthened?   - If no, then pick one: Lack of expertise; Funding; Time constraints; Not a management priority; Other:______________________________________________________ | Yes/No  Yes/No | Yes/No  Yes/No |
|  | **C. Employee Occupational Health / Infection Control programs**?   - If no, then pick one: Lack of expertise; Funding; Time constraints; Not a management priority; Other:______________________________________________________   **D.** As a result of the assessment, have new or existing programs been implemented and/or strengthened?   - If no, then pick one: Lack of expertise; Funding; Time constraints; Not a management priority; Other:_______________________________________________________ | Yes/No  Yes/No | Yes/No  Yes/No |
|  | **E. Safety Equipment, Calibration, & Maintenance programs:**  **E.1.** Assessment of **Personal Protective Equipment (PPE):**  **E.2.** As a result of the assessment, have new or existing programs been implemented and/or strengthened**?**   - If no, then pick one: Lack of expertise; Funding; Time constraints; Not a management priority; Other:______________________________________________________   **E.3.** Assessment of **Large safety instrument:** (Ex: Biological safety Cabinets, Autoclaves, Centrifuges, etc.)?  **E.4.** As a result of the assessment, have new or existing programs been implemented and/or strengthened?   - If no, then pick one: Lack of expertise; Funding; Time constraints; Not a management priority; Other:_______________________________________________________ | Yes/No  Yes/No | Yes/No  Yes/No |
|  | **F.** **Building & Facility safety evaluation?** (Ex. Mechanical Sys, Ventilation Sys, Electrical   - If no, then pick one: Lack of expertise; Funding; Time constraints; Not a management priority; Other: ________________________________________________________   **G.** As a result of the assessment, have new or existing programs been implemented and/or strengthened?   - If no, then pick one: Lack of expertise; Funding; Time constraints; Not a management priority; Other: ________________________________________________________ | Yes/No  Yes/No | Yes/No  Yes/No |
|  | **H.** **Building & Facility safety evaluation?** (Ex. Mechanical Sys, Ventilation Sys, Electrical)   - If no, then pick one: Lack of expertise; Funding; Time constraints; Not a management priority; Other: ________________________________________________________   **I.** As a result of the assessment, have new or existing programs been implemented and/or strengthened?   - If no, then pick one: Lack of expertise; Funding; Time constraints; Not a management priority; Other: ________________________________________________________ | Yes/No  Yes/No | Yes/No  Yes/No |
|  | **J. Chemical Management program evaluation?**   - If no, then pick one: Lack of expertise; Funding; Time constraints; Not a management priority; Other: ________________________________________________________   **K.** As a result of the assessment, have new or existing programs been implemented and/or strengthened?   - If no, then pick one: Lack of expertise; Funding; Time constraints; Not a management priority; Other: ________________________________________________________ | Yes/No  Yes/No | Yes/No  Yes/No |
|  | **L. Waste Management program evaluation?**   - If no, then pick one: Lack of expertise; Funding; Time constraints; Not a management priority; Other: ________________________________________________________   **M.** As a result of the assessment, have new or existing programs been implemented and/or strengthened?   - If no, then pick one: Lack of expertise; Funding; Time constraints; Not a management priority; Other: ________________________________________________________ | Yes/No  Yes/No | Yes/No  Yes/No |
|  | **N. Principles of laboratory ‘biosecurity’ evaluation?**   - If no, then pick one: Lack of expertise; Funding; Time constraints; Not a management priority; Other: ________________________________________________________   **O.** As a result of the assessment, have new or existing programs been implemented and/or strengthened?   - If no, then pick one: Lack of expertise; Funding; Time constraints; Not a management priority; Other: ________________________________________________________ | Yes/No  Yes/No | Yes/No  Yes/No |
|  | **P. Transport of Infectious Substances evaluation?**   - If no, then pick one: Lack of expertise; Funding; Time constraints; Not a management priority; Other   **Q.** As a result of the assessment, have new or existing programs been implemented and/or strengthened?   - If no, then pick one: Lack of expertise; Funding; Time constraints; Not a management priority; Other :________________________________________________________ | Yes/No  Yes/No | Yes/No  Yes/No |
|  | **R. Radiation safety programs evaluation?**   - If no, then pick one: Lack of expertise; Funding; Time constraints; Not a management priority; Other: ________________________________________________________   **S.** As a result of the assessment, have new or existing programs been implemented and/or strengthened?   - If no, then pick one: Lack of expertise; Funding; Time constraints; Not a management priority; Other: ________________________________________________________ | Yes/No  Yes/No | Yes/No  Yes/No |
|  | **T. Employee training programs review?**   - If no, then pick one: Lack of expertise; Funding; Time constraints; Not a management priority; Other: ________________________________________________________   **U.** As a result of the assessment, have new or existing programs been implemented and/or strengthened?   - If no, then pick one: Lack of expertise; Funding; Time constraints; Not a management priority; Other: ________________________________________________________ | Yes/No  Yes/No | Yes/No  Yes/No |
| 10 | Does each laboratory have a ‘Safety Manuals and SOPs’ that are readily assessable to employees? | Yes/No | Yes/No |

**III. Change in Results and Processes**

Please select your response to the questions below and provide comments in the space provided.

|  | **Question** |  |  |
| --- | --- | --- | --- |
| 11 | Has a **strategy or plan/s** been developed to address and implement the above laboratory safety programs, as a result of the above evaluations?  If no, indicate which safety programs have not been addressed and why: ____ _______________________________________________________________ | Yes/No | Yes/No |
| 12 | Has management agreed to an **incremental strategy/plan** to address longer term improvements? | Yes/No | Yes/No |
| 13 | Has management provided **appropriate staffing** for implementation the above safety program?  If no, indicate which safety programs have not been addressed and why: ____ _______________________________________________________________ | Yes/No | Yes/No |
| 14 | Has management agreed to provide **annual funding** to implement the above safety programs & activities?  If no, indicate which safety programs have not been addressed and why: ____ _______________________________________________________________ | Yes/No | Yes/No |
| 15 | Has management provided appropriate **facilities and ancillary support** to implement the above safety programs & activities?  If no, indicate which safety programs have not been addressed and why: ____ _______________________________________________________________ | Yes/No | Yes/No |
| 16 | Has the institution and laboratories have developed a **schedule process to re-evaluate safety and progress at defined intervals**? (Ex. Quarterly, Semi-annual, Annual meetings or assessments).  If no, indicate which safety programs have not been addressed and why: ____ _______________________________________________________________ | Yes/No | Yes/No |
| 17 | Have new or existing biosafety programs increased compliance with:   - Local and national safety policies and regulations?   If no, indicate which safety programs have not been addressed and why: ____   - Laboratory accreditation efforts?   If no, indicate which safety programs have not been addressed and why: ____ _______________________________________________________________ | Yes/No | Yes/No |
|  |  | Yes/No | Yes/No |

**IV. Successes and Challenges**

| Please answer **YES** or **NO** to each question below and provide brief comments. | | | | | |
| --- | --- | --- | --- | --- | --- |
|  | **Question** | **Your Answer** | | **Comment** | |
| 18 | How motivated were you to apply the skills you learned during the course to implement changes in your country? |  |  | Rate yourself: 1-5 (5 being highest)  1 2 3 4 5 | |
| 19 | Did you conduct any innovative projects to improve your national or institution’s laboratory safety programs? Please describe any innovations in less than 200 words | Yes | No |  | |
| 20 | How accessible were resources to you for implementing the changes at your institution?  Please specify if any limitation:_______________________________________ |  |  | Rate yourself: 1-5 (5 being 100% accessible)  1 2 3 4 5 | |
| 21 | Was there a person who was most instrumental in providing a positive workplace environment to transfer/implement the learning from the course (specify)? | Yes | No | MOH Director |  |
|  |  |  |  | Organizational Management |  |
|  |  |  |  | Laboratory Manager/Supervisor |  |
|  |  |  |  | Donor |  |
|  |  |  |  | Implementing Partner (IP) |  |
|  |  |  |  | Others ________________________ |  |
| 22 | Please describe your top 3 challenges each during the development of national lab commodities strategic plan in your country (keep very brief) | 1.  2.  3. | | | |

**V. Recommendations**

| How can this course be improved? |  |
| --- | --- |
| Suggested topics or sections for future course: |  |
